# The demographics of Tolosa-Hunt syndrome in Qatar

**DOI:** 10.1016/j.ensci.2021.100359

**Published:** 2021-07-22

**Authors:** Fateen Ata, Zohaib Yousaf, Suresh Nalaka Menik Arachchige, Saman Rose, Awni Alshurafa, Bassam Muthanna, Ammara Bint I Bilal, Ahmed El Beltagi, Muhammad Zahid

**Affiliations:** a Department of Internal Medicine, Hamad General Hospital, Hamad Medical Corporation, Doha, Qatar; b Department of Radiology, Hamad General Hospital, Hamad Medical Corporation, Doha, Qatar; c Department of Neuroscience and Neuroradiology, Hamad Medical Corporation, Doha, Qatar; d Weill-Cornel Medicine, Qatar

**Keywords:** Tolosa Hunt syndrome, THS, Cavernous sinus, Ophthalmoplegia, Third nerve palsy

## Abstract

**Background:**

Tolosa Hunt syndrome (THS) is a rare disease that manifests mainly as painful unilateral ophthalmoplegia. It is caused by an inflammatory process of unknown aetiology within the cavernous sinus with a rare intracranial extension. The International Classification of Headache Disorders (ICHD)- 3 diagnostic criteria aids in its diagnosis. There is limited literature on its varied presentations, diagnosis, and management. Steroids are used in the treatment of THS with varied success.

**Methods:**

We conducted a single-center-retrospective-study and included all patients admitted with a diagnosis of THS from January 2015 to December 2020. Descriptive and summary statistics were used to describe the study cohort's socio-demographic parameters.

**Results:**

Among 31 THS patients (predominantly Asians (18) and Arabs (9)), visual disturbance was commonest presenting complaint. Third-nerve paralysis was seen in 70.9% cases. Magnetic-resonance-imaging (MRI) was abnormal in 64.5%. 93.5% patients received steroids, with a response-rate of 70.9% and a recurrence-rate of 9.7%. A previous history of THS and female gender were associated with recurrence (*p*-value 0.009 and 0.018). Recurrence was seen in 66.7% fully recovered and 33.3% partially recovered cases (p-value 0.04). Among the benign and inflammatory subtypes of THS, the ICHD-3 criteria were applicable in 85% of inflammatory THS.

**Conclusions:**

THS is a rare disease with ethnic variation in presentation and response to treatment. In our cohort female gender and a previous history of THS were associated with recurrence. ICHD-3 diagnostic criteria had a higher validity in our patients compared to prior studies, especially among the inflammatory THS.

## Highlights

• We represent the largest cohort of THS patients from the Middle eastern population.
• In our cohort, medium dose steroids (60 mg daily) had a good recovery rate (71%).
• Female gender and a prior history of THS were associated with recurrence.
• Recurrence rate was low in our cohort compared to international trends (9.7%).

## 1 Introduction

Tolosa-Hunt syndrome (THS) is one of the rare differentials of painful ophthalmoplegia. The exact prevalence remains unknown; however, the annual incidence is estimated to be as low as one case per million population [1]. The prevalence or incidence of THS remains unknown and is also not mentioned in the portal for rare diseases and orphan drugs [2]. THS is characterized by granulomatous inflammation of the cavernous sinus. Common clinical manifestations of THS include unilateral orbital pain and ipsilateral third nerve palsy. Diagnosis mainly relies on the exclusion of other causes of third nerve paralysis. International Headache Society (IHS) has proposed THS diagnostic criteria, including a unilateral headache, granulomatous inflammation of the cavernous sinus, superior orbital fissure or orbit, and a causal relationship of the inflammation with the headache [3]. Steroids were introduced in the management of THS by Hunt in 1961; however, to date, there is no clear evidence for the optimal dose and duration of the treatment [4]. Steroids are currently recommended for patients who fulfil both the clinical and diagnostic criteria [5].

Like other diseases, THS has variations in presentation and clinical course. Although one of the diagnostic criteria components, patients may not have granulomatous inflammation on imaging [6]. Additionally, there is a varied response to the treatment with steroids. This may be attributed to the demographic variance in the patients [7]. THS is a highly under-explored aspect in the Middle East, and the data is limited to a few case reports [[8], [9], [10]]. We have conducted a retrospective study in Qatar and analyzed patients admitted with a diagnosis of THS from 2015 to 2021 in a tertiary care hospital. We studied the cases based on demographics, diagnosis, and clinical course of the disease to provide data regarding THS in the Arab world. Additionally, we also evaluated the applicability of the IHS diagnostic criteria on our patient population.

## 2 Research design and methods

### 2.1 Study design

We have conducted a single-center, retrospective study on patients admitted with a diagnosis of THS at a single tertiary care center from January 2015 – January 2021.

### 2.2 Inclusion criteria

All patients admitted to the tertiary care hospital of Weill Cornell Medicine affiliated-Hamad Medical Corporation, Qatar, with a diagnosis of THS between 2015 and 2021 were included in the study. The patient cohort comprised patients who had no prior neurological disease. The diagnosis of THS was made based on IHS diagnostic criteri into benign (normal imaging) and inflammatory (granulomatous inflammation on imaging). Patients who had the wrong diagnosis coding and those who got an alternate diagnosis in the same admission or follow-ups were excluded from the study.

Data of included patients with THS from 2015 to 2021 were abstracted from electronic records of Hamad Medical Corporation patient data repository (Cerner). Data collected includes demographics such as age, sex, nationality, comorbid conditions, and relevant laboratory investigations at admission, including C reactive protein (mg/L), erythrocyte sedimentation rate (mm/h), and cerebrospinal fluid (CSF) analysis. Data regarding THS symptoms include the presence of a headache, visual disturbance, complete third nerve palsy, partial third nerve palsy, pupils spared or not, fourth cranial nerve involvement, sixth cranial nerve involvement, trigeminal (V1) nerve involvement, side involved, and any prior history of third nerve palsy.

Radiological data includes the Chest X-ray, CT-Brain, MRI, MRA, and MRV head findings. Other data related to diagnosis includes the findings of the biopsy where applicable. Treatment variables include the type of steroid given with the dose and duration. Data related to recovery includes MRI findings post-treatment, whether the diagnosis was changed or maintained in the follow-up (3- and 6-month follow-ups), and recurrence (1 year follow up). Patients were divided into benign THS (no radiological evidence of inflammation) and inflammatory THS (radiological findings consistent with inflammation).

### 2.3 Statistical analyses

Descriptive and summary statistics were used to describe the study cohort's socio-demographic parameters, with continuous variables presented as means (±standard deviation) or median (interquartile range) as appropriate, while categorical variables as numbers (percentages). Shapiro-Wilk test was used to assess the normality of our dataset. Kruskal-Wallis test was used to analyze associations of various variables with outcomes. All data were analyzed using Jamovi version 1.2 (created in 2020, Sydney, Australia) [11].

## 3 Results

### 3.1 Demographics

A total of 31 patients were included in the analysis after application of inclusion and exclusion criterion (Table 1). 11 patients had a benign disease whereas 20 patients had inflammatory THS. The study cohort comprised of 22 (71%) males and 9 (29%) females. The median age was 40 (32–48.5) years. Majority of the patients were Indians (*N* = 10, 32.3%), followed by Nepalese (*N* = 6, 19.4%), Sudanese (*N* = 4, 12.9%), Qataris (*N* = 2, 6.5%), American (*N* = 1, 3.2%), British (N = 1, 3.2%), Egyptian (N = 1, 3.2%), Filipino (N = 1, 3.2%), Indonesian (N = 1, 3.2%), Lebanese (N = 1, 3.2%), Romanian (N = 1, 3.2%), Syrian (N = 1, 3.2%) and Tanzanian (N = 1, 3.2%).

**Table 1:** Demographics of patients admitted with Tolosa Hunt Syndrome in Qatar.

Characteristics	Results (Median with IQR or N with %)
Age (years) (*N* = 31)	40 (32–48.5)
**Gender (*N* = 31)**	
Males	22 (71%)
Females	9 (29%)
**Nationality (*N* = 31)**	
Indian	10 (32.3%)
Nepalese	6 (19.4%)
Sudanese	4 (12.9%)
Qatari	2 (6.5%)
American	1 (3.2%)
British	1 (3.2%)
Egyptians	1 (3.2%)
Filipino	1 (3.2%)
Indonesian	1 (3.2%)
Lebanese	1 (3.2%)
Romanian	1 (3.2%)
Syrian	1 (3.2%)
Tanzanian	1 (3.2%)
**Comorbidities (*N* = 31)**	
Diabetes	5 (16.1%)
Hypertension	8 (25.8%)
Autoimmune disease	1 (3.2%)
Smoker	4 (12.9%)
Prior THS	1 (3.2%)
Previous history 3rd nerve palsy	3 (9.7%)
Other neurological disease	0
**Features at diagnosis (*N* = 31)**	
Headache	25 (80.6%)
Visual disturbance	30 (96.8%)
Complete 3rd nerve paralysis	5 (16.1%)
Partial 3rd nerve paralysis	17 (54.8%)
Sparing of pupils	22 (71%)
Involvement of 4th cranial nerve	8 (25.8%)
Involvement of 6th cranial nerve	17 (54.8%)
V1 nerve involvement	4 (12.9%)
**Side involved (*N* = 31)**	
Right	12 (38.7%)
Left	19 (61.3%)
Laboratory workup	
CRP (mg/L) (*N* = 18)	1.75 (0.92–5.07)
ESR (mm/h) (*N* = 13)	10 (9–25)
**CSF analysis (*N* = 31)**	
Normal	21 (67.7%)
Abnormal	3 (9.7%)
Not done	7 (22.6%)
**CXR**	
Normal	22 (71%)
Abnormal	1 (3.2%)
Not done	8 (25.8%)
**CT-Brain**	
Normal	26 (83.9%)
Abnormal	3 (9.7%)
Not done	2 (6.5%)
**MRI**	
Normal	11 (35.5%)
Abnormal	20 (64.5%)
Not done	0
**MRA**	
Normal	17 (54.8%)
Abnormal	9 (29%)
Not done	5 (16.1%)
**MRV**	
Normal	10 (32.3%)
Abnormal	5 (16.1%)
Not done	16 (51.6%)
Granulomatous inflammation of the cavernous sinus	20 (64.5%)
High intensity ring appearance around the optic nerve	7 (22.6%)
**Biopsy**	
Normal	5 (16.1%)
Abnormal	0
Not done	26 (83.9%)
Steroids given	31 (100%)
Starting dose of steroids (*N* = 29)	60 (60–60)
Duration of steroids (weeks) (*N* = 28)	6.5 (6–10)
**Response to pain**	
Within 48 h (N = 17)	9 (52.9%)
Within 72 h (N = 14)	12 (85.7%)
**Recovery**	
No recovery	4 (12.9%)
Partial recovery	2 (6.5%)
Complete recovery	20 (64.5%)
No follow up	5 (16.1%)
**MRI post treatment**	
Normal	7 (22.6%)
Abnormal	2 (6.5%)
Not done	22 (71%)
Diagnosis changed	0
**Recurrence**	
Recurrence of THS	3 (9.7%)
No follow up	5 (16.1%)
Fits diagnostic criteria	17 (54.8%)

Among our patient population, 25.8% (*N* = 8) had hypertension, 16.1% (*N* = 5) had Type 2 diabetes mellitus, 12.9% (*N* = 4) were smokers, 9.7% (*N* = 3) had a previous history of 3rd nerve palsy, 3.2% had previous history of Tolosa hunt syndrome (*N* = 1) and 3.2% had a history of an autoimmune disease (N = 1). Patients with benign THS were relatively younger compared to inflammatory THS (38.5 ± 15 years and 43.6 ± 12.3 years, respectively).

### 3.2 Clinical details of THS

**Fig. 1:**
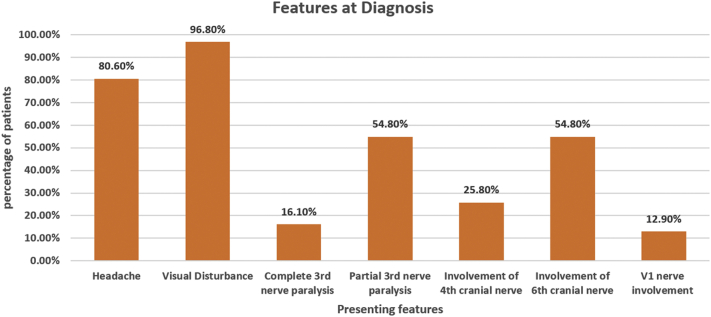
Presenting features of THS.

We found a spectrum of signs and symptoms of THS in our patient cohort ranging from a visual disturbance in 96% to trigeminal (V1) nerve involvement in 12.9%. Other features included headache (80.6%), complete third nerve palsy (16.1%), partial third nerve palsy (54.8%), sixth and fourth cranial nerve paralysis (54.8% and 25.8%, respectively) (Fig. 1). 61.3% (*N* = 19) patients had left-sided THS whereas right-sided THS was seen in 38.7% (*N* = 12). Eighteen patients had a reported C-reactive protein (CRP) level with a median of 1.75 (0.92–5.07) mg/L. ESR was reported in 13 patients with a median of 10 (9–25) mm/h. Cerebrospinal fluid (CSF) was taken for analysis in 24 patients. Only three patients had an abnormal CSF (one had high proteins only, and two had low glucose). Cell counts were normal in all three patients. Chest x-ray was done in 22 patients and was abnormal in 1 patient only with right upper zone opacification. CT brain was abnormal in 3 (9.7%) patients. One patient had a filling defect in the superior sagittal sinus. Another patient had a mild asymmetric thickening of the left optic nerve sheath complex. The third patient had calcifications in basal ganglia and foramen of Monro, along with mucosal thickening in maxillary sinuses bilaterally.

**Fig. 2:**
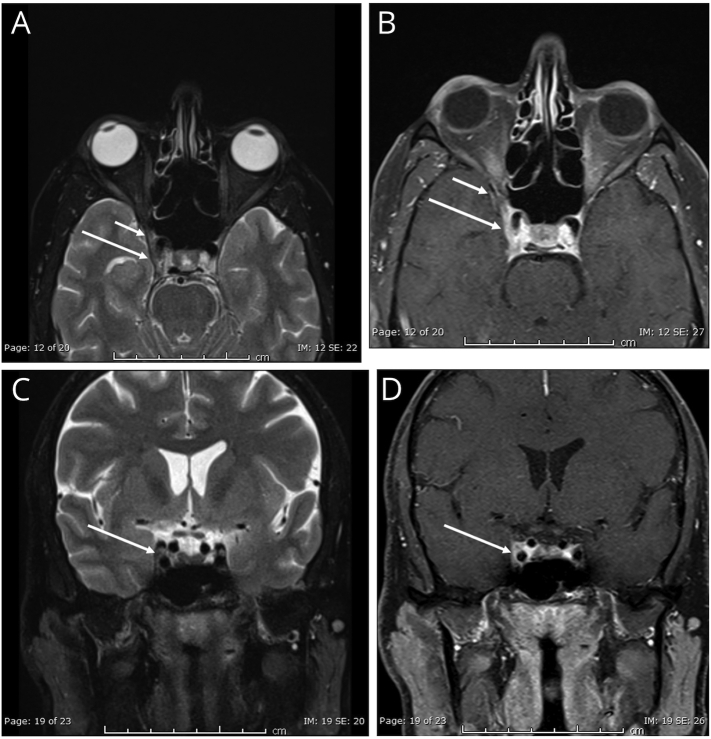
MRI orbits T2 WI, and post I.V. Gadolinium based contrast T1WI, axial orientation (a, and b respectively), and coronal orientation (c, and d respectively), showing T2 intermediate signal and post contrast enhancement increased thickening involving the right cavernous sinus (long arrows in a, b, c, and d), extending to the su*peri*or orbital fissure (short arrows in a, and b).

**Fig. 3:**
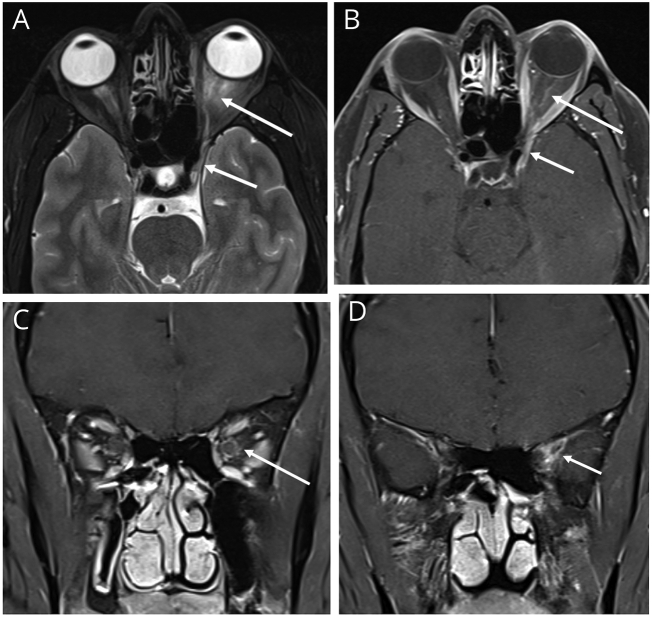
Gadolinium based contrast T1WI, axial orientation (a, and b respectively), and coronal orientation post I.V. Gadolinium based contrast T1WI (c, and d respectively) at the level of intraorbital optic nerve, and at the level of superior orbital fissure respectively, showing involvement of the left optic nerve showing peripheral nerve enhancement and peri optic fat stranding (long arrows in a, b, and c), thickening and mild outward bulge of the left cavernous sinus extending to left orbital apex at superior orbital fissure (short arrows in a, and d).

On the other hand, an MRI scan of the head was abnormal in 20 patients (64.5%). MRA and MRV scans were abnormal in 9 (29%) and 5 (16.1%) patients, respectively. Among the abnormalities seen in the magnetic resonance scans, granulomatous inflammation of the cavernous sinus was present in all cases (*N* = 20), and a high-intensity ring appearance around the optic nerve was seen in 7 (35%) patients. Granulomatous inflammation in the scans was found in the cavernous sinus (*N* = 9, 45%), orbital apex (*N* = 5, 25%), superior orbital fissure (N = 5, 25%), intra-orbital optic nerve, and the dural sinus (*N* = 1, 5% each). A biopsy was performed in 5 (16.1%) patients with normal results. Common radiological findings found in the patients are shown in figures (Fig. 2a, b, c, d showing cavernous sinus enhancement, Fig. 2a, b showing superior orbital fissure involvement, and Fig. 3 showing optic nerve involvement). When comparing the clinical details of benign and inflammatory THS, the median CRP was interestingly higher in benign compared to inflammatory subtype (2.05 vs. 1.45 mg/L). However, the median ESR was higher in the inflammatory subgroup (12 mm/h) compared to benign THS (9.5 mm/h). The frequencies of presenting features were more or less similar in both groups, as shown in Table 2. Visual disturbance was the most common (100% in benign and 95% in inflammatory THS), whereas V1 nerve involvement was the most infrequent in both groups (9.09% and 15% in benign and inflammatory THS, respectively). The left side was involved in 63.6% of benign and 55% of inflammatory THS. Among the inflammatory THS patients, abnormal magnetic resonance angiogram (MRA) and magnetic resonance venogram (MRV) were found in 45% and 25% of cases, respectively. The scans showed cavernous sinus involvement in 9 cases and dural sinus involvement in 1 patient.

**Table 2:** Demographics of patients based on the type of THS (benign and inflammatory THS).

Characteristics	Benign THS (*N* = 11)	Inflammatory THS (N = 20)
Age in years (Mean ± SD)	38.5 ± 15	43.6 ± 12.3
**Features at diagnosis (*N* with %)**		
Headache	10 (90.0%)	15 (75%)
Visual Disturbance	11 (100%)	19 (95%)
Complete 3rd nerve paralysis	2 (18.18%)	3 (15%)
Partial 3rd nerve paralysis	5 (45.45%)	12 (60%)
Sparing of pupils	7 (63.6%)	15 (75%)
Involvement of 4th cranial nerve	2 (18.18%)	6 (30%)
Involvement of 6th cranial nerve	7 (63.6%)	10 (50%)
V1 nerve involvement	1 (9.09%)	3 (15%)
**Side involved (*N* with %)**		
Right	4 (36.6%)	8 (40%)
Left	7 (63.6%)	11 (55%)
Abnormal MRA (N with %)	0	9 (45%)
Abnormal MRA (N with %)	0	5 (25%)
**Laboratory investigations (Median with IQR)**		
CRP (mg/L)	2.05 (1.6–4.38)	1.45 (0.77–5.32)
ESR (mm/h)	9.5 (8.76–25)	12 (9–19)
Steroid treatment		
Starting dose in mg	60 (40–60)	60 (60–77.5)
Duration in weeks	6 (6–12)	7 (6–10)
**Response to pain (*N* with %)**		
Within 48 h	4/8 (50%)	5/9 (55.6%)
Within 72 h	7/8 (87.5%)	5/6 (83.3%)
**Recovery of ophthalmoplegia (*N* with %)**		
No recovery	2 (18.18%)	2 (10%)
Partial recovery	1 (9.09%)	1 (5%)
Complete recovery	6 (54.5%)	14 (70%)
No follow up	2 (18.18%)	3 (15%)
**Recurrence (*N* with %)**		
Recurrence of THS	0	3 (15%)
Fits Diagnostic Criteria (N with %)	0	17 (85%)

### 3.3 Outcomes

**Fig. 4:**
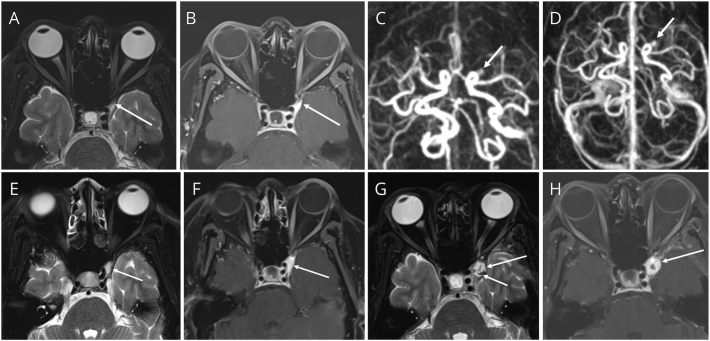
MRI orbits at initial presentation: axial image T2WI (a) TIW post contrast (b) showeing small heterogenous signal on T2WI and intensely enhancing lesion in the anterior left anterolateral cavernous sinus (arrows in a, and b), and dynamic post contrast MR angiography showing opacification in the late arteriographic phase (c), extending into the venous phase (d) (arrows in c, and d). MRI orbits axial image T2WI (e) TIW post contrast (f) showing progression in size (arrows in e, and f). Follow-up MRI follow up after 6 months: T2WI (g) showing further increase in the size, T2 heterogenous bright signal, with intralesional hypointensities (long and short arrows in in g respectively) in keeping with a hemorrhagic component and intense marginal enhancement on T1WI post contrast image (arrow in h).

Steroids were given in 29 patients the patients with a tapering plan. Two patients did not receive steroids and the reason for not initiating steroid therapy was not specified. The median starting dose and duration of steroids were 60 mg and 6.5 (6–10) weeks, respectively. 64.5% (*N* = 20) patients had a complete recovery, and 6.5% (N = 2) patients had a partial recovery. 12.9% (4) patients did not recover and 16.1% (*N* = 5) were lost to follow-up. At the follow-up visits, none of the patients had a change in their diagnosis. Recurrence of THS was found in 3 (9.7%) patients. 24 patients (77.4%) in our cohort fulfilled the International Headache Society (IHS) THS diagnostic criteria. A complete recovery was seen more in inflammatory THS than benign THS patients (70% vs. 54.5%). However, recurrence at 1-year follow-up was seen only in inflammatory THS (Table 2). One of the patients had a progression at a 6-month follow-up in his initial radiological findings of granulomatous inflammation (Fig. 4). All data was analyzed based on recovery and recurrence of THS. A history of previous THS was associated with a partial recovery (*N* = 1, 100%, *p*-value = 0.002) and was also strongly associated with recurrence of THS (N = 1, 100%, p-value = 0.009). Recurrence (*N* = 3) was solely found in females (37.5%, p-value = 0.018). Among the patients who recovered, recurrence was seen in 66.7% and 33.3% of fully and partially recovered patients, respectively (p-value 0.04).

## 4 Discussion

We present one of the most extensive studies on THS from the Middle East with a diverse patient population. We found the International Headache Society (IHS) THS diagnostic criteria applicable to 77.4% of our patients. Steroid treatment had a particularly good clinical response in our cohort (70.9% including complete and partial response). The recurrence rate (at 1 year follow-up) was low (9.7%) with no mortality. The median age (40 years) in our study population is comparable to previously reported data [7,12]. The cohort was male predominant, which is comparable to previous data. However, the difference was more pronounced than previously reported (71% in our study vs. 36.4% and 65% in previous studies) [7,12]. This can be explained by a male dominant population of Qatar due to more male expatriates (around 19 million males compared to 7 million females) [13]. We found lesions to be present mainly on the left side (*N* = 19, 61.3%), whereas 12 (38.7%) patients had a lesion on the right side. This is contrary to what is previously reported. Zhang Et al. reported right laterality in 30.4% and left laterality in 19.6% patients [12].

Clinical features and presentations of patients with THS are variable with a spectrum of commonly encountered signs and symptoms. The most common symptom of THS includes painful ophthalmoplegia with the involvement of third, fourth, or sixth cranial nerve [5]. Previous studies have reported variable frequencies of the clinical presentations of THS. In their cohort of 44 patients, Arthur et al. reported ptosis with ophthalmoplegia as the most common feature (66%), whereas pain was the initial complaint in 90% of patients [14]. We also found that visual disturbance secondary to ophthalmoplegia was the most common presenting complaint (96.8%), followed by headache (80.6%). The most infrequent finding was V1 nerve involvement (12.9%), which is in accordance with the previous trends. Although the 3rd nerve is the most commonly involved (can be up to 73%) in THS according to the literature, we found sixth nerve involvement to be as common as 3rd in our patient population (54.8% each) [12]. There are many rare clinical features reported in association with THS, including paralysis in nerves other than 3rd, 4th, 5th or 6th. It has been reported with 7th nerve paralysis [15]. It has also been reported to present with pituitary findings such as decreased libido, nocturia, and thirst [16]. We did not find any rare associations or presentations of THS in our patients. THS has also been associated with other diseases, such as sarcoidosis, myasthenia gravis, systemic lupus erythematosus, hypertrophic pachymeningitis, and lymphomas, among others [[17], [18], [19], [20]]. None of the patients in our cohort had a prior neurological condition (other than previous THS in one patient). Diabetes and hypertension were common comorbidities, but none of the patients had a concomitant new diagnosis made at presentation with THS.

Before the availability of high-resolution MRI, the diagnosis of THS relied heavily on the clinical response to corticotherapy and biopsy evidence whenever possible [9]. At the time, radiological investigation comprised of cerebral angiography and orbital phlebography [21]. The revised guidelines of the international headache society now include MRI findings in the diagnostic criteria, namely the granulomatous inflammation of the cavernous sinus, superior orbital fissure, or orbit. However, it must be noted that these findings alone do not suffice and must be accompanied by clinical paresis of one or more of the ipsilateral IIIrd, IVth and/or VIth cranial nerves [3]. For patients with high clinical suspicion, MRI is up to 100% sensitive. However, the specificity of MRI is 28.6% [22]. CT scan has a very low diagnostic value in THS and can be completely normal in inflammatory THS [23]. In our study, the CT scan was normal in 83.9% (*N* = 26) of cases. Fifteen of these 26 patients had granulomatous inflammation detected in the MRI scan; hence we concluded that in patients with high clinical suspicion, it is time and cost-effective to perform MRI rather than a CT scan to make a radiological diagnosis.

R. Jain et al., in their description of MRI appearances, reported isointense images on T1W with intense contrast enhancement in the majority of MRI granulomatous lesions. T2W images are known to show variability in terms of signal enhancement, and the same was reported. Their findings corroborated that cavernous sinus is the most common location (85% of the patients), followed by apex and orbit (28%). One of the seven patients had involvement of all three- CS, Apex, and orbit [9]. Our findings correlate with this trend as we found cavernous sinus involvement in the majority of the cases with abnormal imaging (*N* = 9, 45%), followed by orbital apex (*N* = 5, 25%) and superior orbital fissure (N = 5, 25%).

In another retrospective study, Schuknecht et al. described the MRI findings in 15 patients identified by two neuroradiologists and one neuro-ophthalmologist. The physicians were kept unaware of the severity and side of the clinical lesions along with MRI exam timing (initial vs subsequent) to remove bias. They used primary and secondary criteria derived from the initial publications of Tolosa and Hunt et al. Primary criterion relied on findings of the anterior cavernous sinus, namely location, local size increase, and bulging of the dural contour. The secondary criterion included blurring of the dural border in T2w images, involvement of the internal carotid artery, an extension of lesion to the superior orbital fissure, and involvement of the orbital apex and optic nerve [21]. Their study highlights the benefit of utilizing this criterion in diagnosing Tolosa hunt syndrome and stresses that MR findings may be strongly suggestive but not pathognomonic. Follow-up MRI images are required not to miss an alternative diagnosis. 3-D constructive interference in the steady-state (CISS) sequence in MRI is even more promising in diagnosing and following up on this rare disorder as it provides an even higher spatial resolution and is gaining ready accessibility [9].

Majority of the time, the disease is unilateral; however, 3–4% of all cases of THS have demonstrated bilateral involvement. At least one case report also mentions the presence of alternating THS, in which MRI findings mirror clinical alternations [24]. In our patient population, 100% of cases were unilateral, with the right side involved in 12 (38.7%) and the left side in 19 (61.3%) patients. It is not yet established whether the laterality of THS has any impact on its clinical course and outcomes.

There is limited data on a benign variant of THS. In our patient population, 11 patients (35.3%) had a normal MRI scan at diagnosis. The patients with normal imaging with no inflammatory changes have been described to have benign THS with a better recovery rate than the inflammatory type of THS [6]. However, in our cohort, among the ones with benign THS, 18.1% (*N* = 2) did not recover, and two were lost to follow-up. Complete recovery was seen in 8 (88.8%) of patients. Compared to this, patients with inflammatory THS had a lower treatment failure rate (10%, N = 2). This variation in our results can be due to demographical differences in the patients compared to the western population.

Although ICHD-3 criteria require the clinical findings to be supported by abnormal imaging or biopsy findings, a non-inflammatory variant of THS is increasingly being reported with signs and symptoms similar to classical THS, other than a radiological or pathological abnormality [6,25,26]. Given that these patients are diagnosed as THS after careful exclusion of other possibilities and as the response rate to treatment is better than classical THS, the variant should be considered a probable THS in the ICHD-3 diagnostic criteria steroid treatment.

There are no clear guidelines on the role, dose, route, and duration of steroids in THS management, even though steroids have been used and recommended in the management for decades [27]. As there are not many extensive studies on the management of THS, much of the data comes from case reports and series. Zhang et al. reported using steroids in 87.0% of their THS patients, with variable doses and duration [12]. Compared to that, around 94% of our patients were treated with steroids with a median dose of 60 mg and a median duration of 6.5 weeks.

The response rate to steroid treatment in THS varies, and it is still unclear what are the exact determinants of the difference in response rates. Even though the clinical response to steroid therapy is prompt, MRI features can lag and take months to resolve [24]. This should be kept in mind while following a patient with THS. In our cohort, 64.5% had a complete response to steroids with no residual pain and cranial nerve palsy at follow-up. 6.5% of patients had only partial response, i.e., the pain was relieved with residual nerve paralysis. 12.9% of the patients did not have a clinically detectable response to steroids. In comparison, one of the prior studies has reported a 77.7% response rate regarding pain and a partial response to nerve palsy and other THS symptoms in 70%, whereas the treatment failure rate was 30% [7]. The authors concluded that young age could be a risk factor for the recurrence of THS; however, young age was associated more with non-inflammatory THS. We had a very low rate of 1-year recurrence (9.7%) in our patient population, so we could not derive any such conclusions. In a previous THS validation study, Zhang et al. reported pain relief at 72 h in 40% of their patients [12]. We had different results in our patient population. In our cohort, we could not get details of pain relief in all patients as some of them were discharged before 72 h. Among those who had a reported outcome of pain at 72 h (*N* = 14), pain relief was seen in 12 (85.7%). Pain relief at 48 h (*N* = 17) was seen in 9 patients (52.9%). Literature review reveals a very diverse recurrence rate, ranging from 14.2% to 48.6% [9,12,14,23]. However, it is still unclear what are the exact determinants of relapse. Our patients received a higher starting dose and a longer duration (median of 6.5 weeks) which could be a reason for low relapse rates.

Validity of THS diagnostic criteria has a vital role in the accurate diagnosis and management as the condition is mainly a diagnosis of exclusion. Few studies have validated the widely accepted ICHD-3 THS diagnostic criteria. Zhang et al., in their 46 patients, used ICHD -3 beta diagnostic criteria. They reported a validation rate of 52.17% [12]. In another smaller case series, ICHD-3 diagnostic criteria were applied retrospectively to the 10 cases admitted with a THS diagnosis, from which 50% fulfilled the criteria [28]. In our patients, we also used the International Headache Society (IHS) ICHD-3 diagnostic criteria to validate diagnosis [3] (Table 3). 54.8% of our patients did fulfil the diagnostic criteria. The slight variation in the validation can be secondary to the small sample size in both studies, but it can also be due to the ethnic variation of the patient population. This is reflected in our study also where non-inflammatory THS seems to be more prevalent compared to global trends. Although difficult for rare diseases, more extensive studies are required to validate the diagnostic criteria accurately.

**Table 3:** ICHD-3 beta diagnostic criteria ICHD: (International Classification of Headache Disorders).

A. Unilateral headache fulfilling criterion C
B. Both of the following: 1. Granulomatous inflammation of the cavernous sinus, superior orbital fissure or orbit, demonstrated by MRI or biopsy. 2. Paresis of one or more of the ipsilateral third, fourth and/or sixth cranial nerves
C. Evidence of causation demonstrated by both of the following: 1. Headache has preceded paresis of the third, fourth and/or sixth nerves by less than 2 weeks or developed with it. 2. Headache is localized around the ipsilateral brow and eye
D. Not better accounted for by another ICHD-3 diagnosis

Although it is an integral part of the ICHD-3 diagnostic criteria for THS, it has been estimated that around 50% of patients may not have any radiological or pathological evidence of inflammation [29]. Hung et al. compared features of benign and inflammatory types of THS in a cohort of 49 patients. They found the prevalence of benign disease to be higher (57.1%). Our findings are different, with a point prevalence of benign THS at 35.4%. Another difference is in the median age group among the two groups. The previous study showed a higher age group having a benign form of the disease (56.4 ± 12.3 years vs. 46.7 ± 19.4 years). In our study, the mean age of patients with benign THS was 38.5 ± 15 years, compared to 43.6 ± 12.3 years in inflammatory THS. ESR was normal in both subgroups. This can be explained by Qatar's relatively younger labour population, which makes a considerable proportion of the total population. However, when compared among the subtypes, the median ESR was higher in inflammatory THS in our dataset, consistent with the trend found by Hung et al. [29]. As in the previous study, a higher combined recovery rate was seen in inflammatory THS (75% vs. 95.7% in the previous study). We found the treatment failure rate to be higher in benign THS (18.18%) compared to inflammatory THS (10%). However, loss of follow-up in 5 patients could have caused these unexpected changes. One of the most striking differences was seen in the validity of the diagnostic criteria. However, this variation was expected as radiological or pathological evidence of inflammation is an essential part of the ICHD-3 criteria. None of the patients with benign THS fulfilled the criteria, whereas it was applicable in 85% of cases in the inflammatory subtype. We can conclude that for benign THS, a careful exclusion of other causes, partial fulfilment of the ICHD-3 criteria (if the patient fulfils points other than B in Table 3) and an adequate response to steroids is sufficient to diagnose the patients as probable THS or benign THS.

Our study has some limitations inherent to retrospective chart reviews. Firstly, due to the rarity of the disease, our sample size was small. A larger sample size could have increased the validity and generalizability of our results. Secondly, we had 16.1% of patients lost to follow-up, which could have impacted the results. This study's principal strength lies in its novelty for being the first extensive original study on Tolosa hunt syndrome from the Middle East with a vast ethnic diversity in the patient population.

## 5 Conclusion

Tolosa Hunt syndrome is a rare disease with unclear guidelines on treatment. We presented the largest THS cohort from the Middle East with an excellent response to medium dose steroids. ICHD-3 diagnostic criteria had a higher validity in our patient cohort. In our cohort, the female gender and a previous history of THS were associated with recurrence. The validity is considerably good in inflammatory THS, whereas it does not apply to benign THS to confirm the diagnosis. More extensive studies are needed to formulate diagnostic criteria for non-inflammatory or benign THS patients so that diagnosis and treatment are not delayed, especially in the parts of the world where benign subtype is prevalent.

## Funding

None.

## Ethics declaration

This work is original, has not been, and is not under consideration for publication in any other Journal. All authors have reviewed and approved the final version of the manuscript. The study was approved by the Medical Research Centre (MRC) Qatar (MRC-04-21-041).

## Consent to participate

Informed consent was not required, as this study was a retrospective data review of medical records.

## Consent for publication

Informed consent was not required, as this study was a retrospective data review of medical records.

## Availability of data and material

Available upon request.

## Code availability

Available upon request.

## Author contribution

*FA*: Methodology, literature review, data collection and interpretation, manuscript writing, critical review and revisions in the manuscript, funding acquisition.

*ZY*: literature review, data analysis and interpretation, manuscript writing, critical review, and revisions in the manuscript.

*SA, AA, SR, BM*: literature review, data collection.

*AB, AE*: literature review, manuscript writing and critical review, preparation of the radiological images.

*MZ*: Conceptualization, supervision, critical review, and revisions in the manuscript.

*All authors*: Review and approval of the final manuscript.

## Declaration of Competing Interest

None of the authors have any conflict of interest to disclose.
